# Identification of an evolutionary conserved structural loop that is required for the enzymatic and biological function of tryptophan 2,3-dioxygenase

**DOI:** 10.1038/srep39199

**Published:** 2016-12-20

**Authors:** Helen Michels, Renée I. Seinstra, Joost C. M. Uitdehaag, Mandy Koopman, Martijn van Faassen, Céline N. Martineau, Ido P. Kema, Rogier Buijsman, Ellen A. A. Nollen

**Affiliations:** 1 European Research Institute for the Biology of Aging, University of Groningen, University Medical Center Groningen, Laboratory of Molecular Neurobiology of Aging, The Netherlands; 2 Netherlands Translational Research Center B.V., Oss, The Netherlands; 3 University of Groningen, University Medical Center Groningen, Department of Laboratory Medicine, The Netherlands

## Abstract

The enzyme TDO (tryptophan 2,3-dioxygenase; TDO-2 in *Caenorhabditis elegans*) is a potential therapeutic target to cancer but is also thought to regulate proteotoxic events seen in the progression of neurodegenerative diseases. To better understand its function and develop specific compounds that target TDO we need to understand the structure of this molecule. In *C. elegans* we compared multiple different CRISPR/Cas9-induced *tdo-2* deletion mutants and identified a motif of three amino acids (PLD) that is required for the enzymatic conversion of tryptophan to N-formylkynurenine. Loss of TDO-2’s enzymatic activity in PDL deletion mutants was accompanied by an increase in motility during aging and a prolonged lifespan, which is in line with the previously observed phenotypes induced by a knockdown of the full enzyme. Comparison of sequence structures suggests that blocking this motif might interfere with haem binding, which is essential for the enzyme’s activity. The fact that these three residues are situated in an evolutionary conserved structural loop of the enzyme suggests that the findings can be translated to humans. The identification of this specific loop region in TDO-2–essential for its catalytic function–will aid in the design of novel inhibitors to treat diseases in which the TDO enzyme is overexpressed or hyperactive.

Genetic variation, environmental factors and aging can lead to malfunctioning or hyperactive proteins, thereby provoking reactions in the cell that might underlie certain diseases. Understanding the structure of such proteins is required when aiming to generate disease-specific compounds that interfere with the protein’s function.

One such protein, that has recently attracted the attention as a potential therapeutic target, is tryptophan 2,3-dioxygenase (TDO in humans, TDO-2 in *C. elegans*) (e.g. ref. 1 and summarised in refs 2 and 3), a cytosolic enzyme that converts tryptophan into formyl kynurenine, thereby catalysing the first rate-limiting step of the kynurenine pathway^4^. In animal models, inhibition or depletion of TDO has been shown to suppress disease phenotypes and to prolong lifespan^5^,^6^,^7^,^8^,^9^. This is thought to be mediated by inhibition of different tryptophan-related pathways that underlie the diseases. For example, since the kynurenine pathway is used to convert 95% of cellular tryptophan, it greatly influences the availability of this amino acid for other tryptophan-related metabolic pathways, including, but not limited to, the production of serotonin^10^. A lack of serotonin has, among others, been associated with depression disorders, which are usually treated with selective serotonin re-uptake inhibitors (SSRIs) to increase serotonin levels (summarised in ref. 11). Secondly, the kynurenine pathway produces metabolites called kynurenines, and an imbalance of certain kynurenines such as kynurenic acid, 3-hydroxykynurenine and quinolinic acid is thought to have profound effects on brain function, may cause a range of neuron-associated diseases (e.g. ref. 12 and summarised in ref. 13) and was recently found being associated with diabetes type 2^14^. Thirdly, overexpression of TDO in certain cancers is thought to increase the tumour’s resistance to the T-cell-mediated immune response which might be dependent on tryptophan in the microenvironment^15^. Since the majority of the phenotypes described above are accompanied by overexpression or hyperactivity of TDO, it is possible that inhibiting the activity of TDO would increase tryptophan and subsequently serotonin levels and control the production of kynurenines.

The development of such compounds requires a thorough understanding of the structure of TDO. Fortunately, the conversion of tryptophan by TDO is evolutionarily conserved from yeast to humans^16^, which means that studies in other organisms may provide useful information on both its structure and function. For example, from such studies we know that TDO is mainly present as homodimers that can form tetramers to be enzymatically active^4^ and that the enzymatic reaction it catalyses is haem and oxygen-dependent^17^,^18^. The haem and tryptophan binding sites in human TDO are also known, and the enzyme’s 3D structure was first described by Forouhar and colleagues for TDO in the bacterium *X. campestris*^19^. While the crystal structure of human TDO has now also been reported^20^, it still remains to be determined which specific amino acids in the TDO polypeptide chains are critical for its enzymatic activity.

We created multiple different CRISPR/Cas9-induced deletion mutants of *tdo-2* in the nematode *C. elegans*. A comparison of these mutants revealed that three amino acid residues in the loop that is close to the haem moiety are essential for the enzymatic activity of TDO-2. The effect of the deletion of those three amino acid residues was as severe as that seen for larger truncations of the enzyme. We also found that the loop structure in which these three amino acid residues reside, is evolutionary conserved in human.

These findings will aid in the development of very specific inhibitors of TDO to intervene with certain disorders.

## Results and Discussion

**Figure 1:**
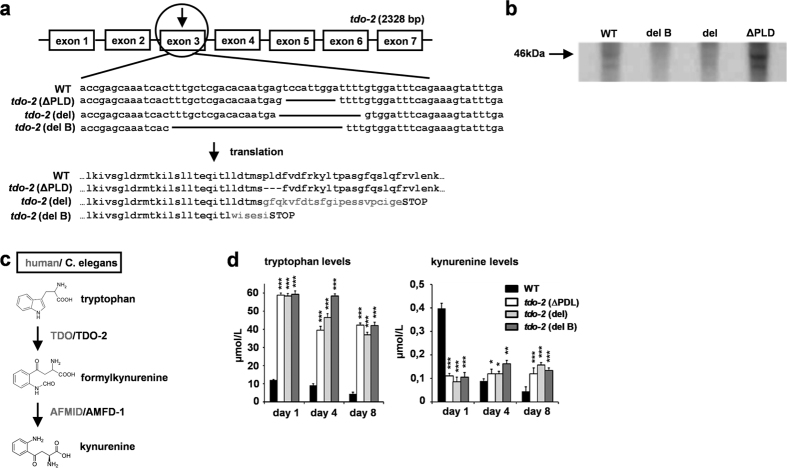
Deletion of PLD sequence impairs conversion of tryptophan by TDO-2. **(a)** CRISPR/Cas9-induced deletions in exon 3 of the *tdo-2* gene. **(b)** Immunodetection of the TDO-2 enzyme in worm lysates from day 4 of adulthood (Cropped image, for full length blot see Supplementary Fig. 1a). **(c)** Schematic representation of TDO-mediated conversion of tryptophan to kynurenine in humans and *C. elegans.* **(d)** Tryptophan and kynurenine levels in worm lysates. Error bars in all panels = SEM. Statistics for all panels, one-way ANOVA with post-hoc Bonferroni (comparison with WT): * < 0.05; ** < 0.01; *** < 0.001.

We mutated *tdo-2* in *C. elegans* with the help of the CRISPR/Cas9 method. To increase the chances of generating truncation mutants we chose a target sequence that was situated the closest to the transcription start. The CRISPR/Cas9 system induces double strand breaks at the targeted sequence. Mistakes during the repair process can lead to mutations, mainly deletions. We got three different mutants with deletions in exon 3 (Fig. 1a), namely *tdo-2 (ΔPLD*), which lacked 9 bp, *tdo-2 (del*), which lacked 14 bp, and *tdo-2 (del B*), which lacked 28 bp. We predicted that the *tdo-2 (del*) and *tdo-2 (del B*) deletions led to a frame shift during translation, resulting in an early STOP codon. Indeed, upon immunoblotting we were unable to detect complete TDO-2 proteins in these mutants (Fig. 1b). However, the mutant with the 9 bp deletion (*tdo-2 (ΔPLD*)) produced a protein that was probably missing exactly three amino acid residues (Fig. 1a) but that was still detectable with the anti-TDO-2 antibody (Fig. 1b).

We then determined whether the deletions resulted in complete knockout mutations. Figure 1c illustrates the first part of the kynurenine pathway whereby TDO (TDO-2 in *C. elegans*) converts tryptophan into formylkynurenine, which is further processed to kynurenine. To this end, we measured tryptophan and kynurenine levels in *C. elegans* lysates. Surprisingly, we saw higher tryptophan levels and reduced kynurenine levels not only in the samples from animals whose TDO-2 proteins had large truncations, but also in the samples from the *ΔPLD* mutants, suggesting that the three amino acids missing in this mutant are essential for tryptophan conversion by TDO-2 (Fig. 1d).

**Figure 2:**
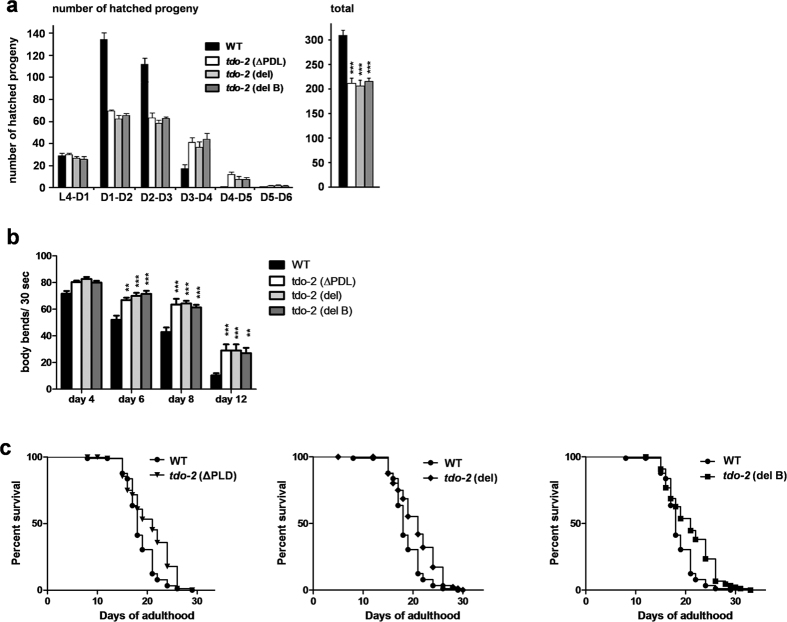
PLD motif in TDO-2 is required for regulation of reproduction, motility and lifespan. **(a)** Reproductive behaviour. **(b)** Motility. Results of interaction by two-way ANOVA considering genotype and time: p < 0.001, p < 0.001, p 0.5205. **(c)** Survival curve of wild type and *tdo-2* mutated animals. Error bars in all panels = SEM. Statistics for all panels (comparison with WT): ** < 0.01; *** < 0.001.

We then checked if the phenotypes of the mutants were the same as those described previously when non-mutated animals were treated with *tdo-2* RNAi^8^. Indeed, all three mutants produced fewer hatched progeny and had an extended reproductive lifespan (Fig. 2a). They also had increased motility (Fig. 2b and Supplementary Fig. S1). Comparing the relationship between time and genotype, the results suggest that differences in motility start to appear at day 4 of adulthood and become more consistent at higher ages, suggesting an age-dependent or accumulative effect. All three mutants show similar results (see legends of Fig. 2b and Supplementary Fig. S1). Finally, the different mutations all similarly extended lifespan (Fig. 2c). To note, the lifespan extension in the three different *tdo-2* mutants was similar to each other but less when compared to the lifespan extension by RNAi knockdown starting after development^8^ and also less robust (Supplementary data Table S2). This suggests that these germline mutations in *tdo-2* may have unknown additional developmental and health consequences that influence lifespan. Still, taken together our results show that the 9 bp ΔPLD deletion is just as effective in mediating TDO-2-specific phenotypes as the removal of almost the entire protein, as it is the case for the *tdo-2*(*del*) and *tdo-2*(*del B*) mutants, indicating that it is an inactivating mutation. The results also suggest that loss of enzymatic activity of TDO-2 is responsible for the observed phenotypes.

**Figure 3:**
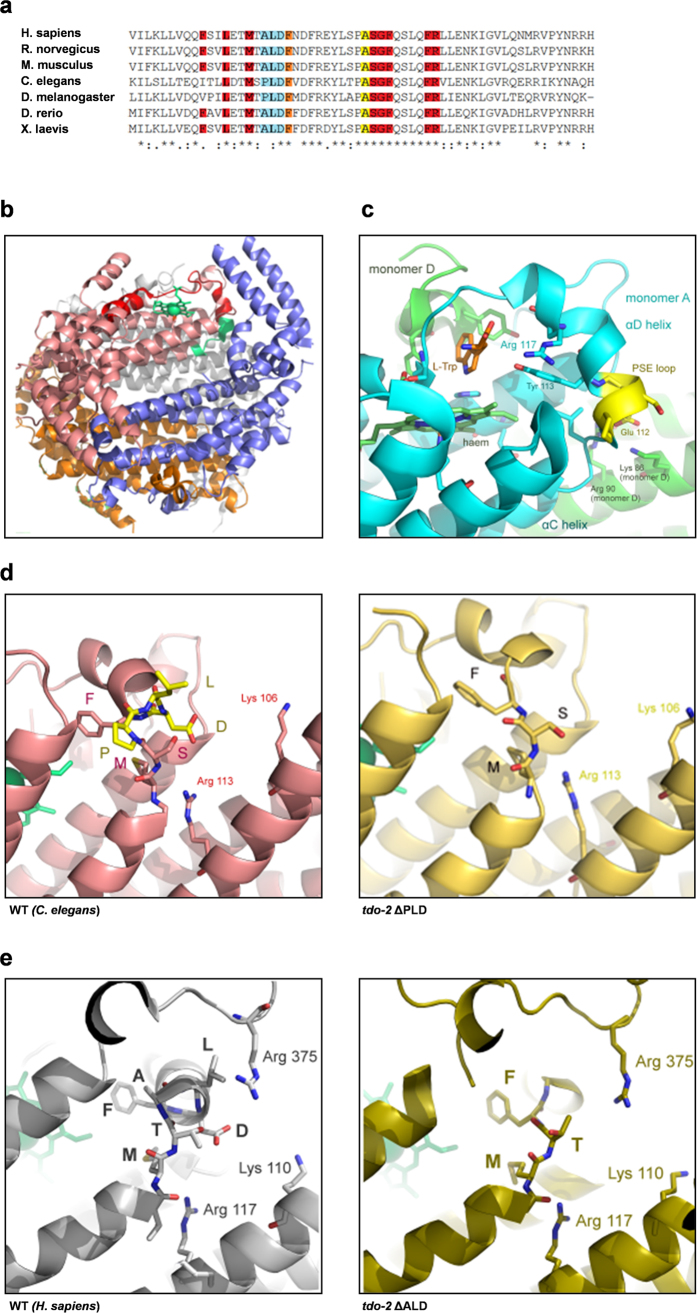
Deletion of PLD motif in TDO-2 is predicted to destabilise the interaction with haem which is essential for enzymatic activity. **(a)** Part of a multiple sequence alignment of TDO of different organisms and *C. elegans* TDO-2; yellow = tryptophan binding site, red = haem binding site, orange = binding site for haem and tryptophan, blue = residues missing in mutant *tdo-2 (ΔPLD*)**. (b)** X-ray structure of the human TDO tetramer (PDB identifier 4PW8, separate monomers in light red, blue, grey and orange), in which a haem group has been added to the model according to the 3D structure of TDO from *X. campestris* (green, PDB identifier NW7). Positioned nearby the haem, also in green, is the loop of residues MSPLDF which includes the PLD residues missing in the *tdo-2 (ΔPLD*) mutant. Amino acids on the C-terminal side of this loop are indicated in red. **(c)** Enlarged image of the X-ray structure of *X. campestris* TDO which contains haem (green) and the substrate L-Trp (orange, PDB identifier 1NW8)^19^. The PSE motif (yellow) is equivalent to the PLD motif in *C. elegans*. Through Glu 112, the motif interacts with monomer D of the tetramer, whereas through Tyr 113 and Arg 117, it forms part of the TDO active site. **(d)** Left: Enlarged image of MSPLDF loop in wild type *C. elegans* TDO-2. Right: Enlarged image of MSPLDF loop in *C. elegans tdo-2 (ΔPLD*) mutant. **(e)** Left: Enlarged image of MTALDF loop in wild type human TDO. Right: Enlarged image of MTALDF loop in human *tdo (ΔALD*) mutant.

Next, to determine why these three amino acid residues might be essential for enzyme activity, we studied the sequence and structure of the TDO-2 molecule. When we aligned the sequences of TDO of different organisms with that of *C. elegans* we found that human and *C. elegans* show a sequence identity of 48.4%. Figure 3a depicts a part of the amino acid sequences, highlighting the residues involved in tryptophan binding (yellow) and haem binding (red) as described by Forouhar *et al*.^19^. Residues forming part of binding sites for both tryptophan and haem are shown in orange (The complete sequence alignment is shown in Supplementary Fig. S3). The sequence comparison shows a strong homology around the site of the PLD deletion (Fig. 3a, highlighted in blue). Whereas the frameshift mutants are lacking most of the tryptophan and haem-binding sites (everything downstream of the PLD site), the PLD mutation is located in a region surrounded by binding sites for tryptophan and haem.

In order to understand the implications of the PLD deletion, we then studied 3D structures. Because the X-ray structure of *C. elegans* TDO-2 is not known, we generated a model based on the 3D structure of human TDO (ref. 20 and Fig. 3b). When enzymatically active, TDO forms a tetramer, as illustrated in the structure depicted in Fig. 3b (monomers in grey, orange, blue and light red). The structure shows that the sequence has a flexible loop (MSPLDF-motif, depicted in green) that connects two alpha-helices that together interact with the haem moiety (green), which is important for the catalytic activity of TDO (ref. 19 and Fig. 3b). This loop is enlarged in Fig. 3c to show the interactions between different TDO monomers that stabilize the final enzyme structure as well as the binding to tryptophan (orange) and haem (green, Fig. 3c).

The *tdo-2 (del*) and *tdo-2 (delB*) mutant proteins are missing the entire ‘lid’ of the catalytic site, which is found on the C-terminal side of the MSPLDF loop (indicated red in Fig. 3b), explaining the lack of catalytic activity and the increased levels of tryptophan in these mutant animals.

We also generated a model for the PLD deletion mutant. The PLD deletion leaves the TDO-2 structure largely intact (Fig. 3d, right-hand panel). However, it shortens the MSPLDF loop (compare enlarged images of WT and *tdo-2 (ΔPLD*) models in Fig. 3d, left and right-hand panels) and removes Asp135 (signified by the D in PLD). This Asp135 residue interacts with lysine (Lys106) and arginine (Arg113) on a neighbouring chain and, the absence of such interaction most likely destabilises the protein’s quaternary structure (Fig. 3b). The PLD deletion also removes Pro133 (signified by the P in PLD), and proline is an amino acid residue known to provide rigidity to turns in protein structures. Based on the important structural functions of these two amino acids, we suggest that the PLD deletion introduces too much flexibility in the MSPLDF loop connecting helix αC and αD of the *C. elegans* TDO-2 structure (nomenclature of the alpha helices according to ref. 19) (Fig. 3c). Furthermore, as demonstrated in the *X. campestris* TDO X-ray structure^19^, the binding of tryptophan to an Arg in the αD helix by salt bridges is key to substrate-enzyme binding (Arg 117 in X. *campestris* as indicated in Fig. 3c, which is equivalent to Arg140 in *C. elegans* and Arg 144 in *H. sapiens*). Subtle disturbances in the positioning of this helix could be the main cause of the protein’s loss of enzymatic activity. In support of this, Dolušić *et al*.^21^ have shown that TDO inhibitors 680C91 and LM10 both strongly interact with the αD helix, thereby emphasizing the importance of our finding for the design of novel specific TDO inhibitors^21^.

Upon studying the conservation of the PLD sequence in several species, we noted that in humans and many other species the proline is replaced by an alanine (resulting in ALD, Fig. 3a). Therefore, a model of human TDO with an ALD deletion (hTDO (ΔALD)) (Fig. 3e) was generated as well. The ALD deletions shortens the loop connecting the αC and αD helices similar to the PLD deletion in the *C. elegans* enzyme. Loss of the aspartate (D) leads to a loss of connections to Arg 375, Lys 110 and Arg 117 in the neighbouring monomer, resulting in expected loss of the stability of the quaternary structure. Though the alanine in ALD does not provide intrinsic loop stability as the proline in PLD does, it is part of the N-terminal moiety of an α-helix. Disruption of this N-terminus through deletion of the ALD sequence will lead to destabilization of the entire helix and will impact the interaction of Arg 117 (X. *campestris* numbering, Arg140 in *C. elegans* and Arg 144 in *H. sapiens*) with the substrate (Fig. 3c–e). Deletion of the ALD motif will, therefore, likely abrogate activity in human TDO and other ALD containing orthologs.

Of the three CRISPR/Cas9-induced mutations in the *tdo-2* gene studied here, two resulted in truncated proteins (early stop codon mutations). While the third mutant was lacking only three amino acids (PLD motif), it also had deficits when considering the conversion of tryptophan to kynurenine and a concomitant increase in motility and a prolonged lifespan. Thus, we have identified a stretch of amino acids in the TDO polypeptide chain that is situated in an evolutionary conserved loop that appears to be critical for this protein’s enzymatic activity.

The early stop codon mutations seem to distort the top half of the active site of TDO-2, preventing binding of tryptophan and haem. Depletion of the PLD motif introduces flexibility in the loop connecting the αD and αC helixes, thereby likely disturbing the αD helix and destabilizing the quaternary structure of the TDO-2 complex.

Interestingly, the loop containing the ALD motif of TDO has never before been identified as being crucial for enzyme activity. This finding will help to develop a new family of TDO-specific inhibitors that can target and destabilise this particular loop region, with the overall aim of treating TDO hyperactivity and tryptophan-associated diseases.

## Methods

### Media and strains

Animals were maintained at 20 °C on Nematode Growth Medium (NGM) and fed with *E. coli* OP50.

Animals were age synchronised by hypochlorite treatment. The following strains were used in this study: Wild type N2, *tdo-2 (del B*) = OW715 *tdo-2 (zg216*)*III; tdo-2 (del*) = OW716 *tdo-2 (zg217*)*III; tdo-2 (ΔPLD*) = OW717 *tdo-2 (zg218*)*III*.

### CRISPR/Cas9-induced mutagenesis

The target sequence was chosen as described in ref. 22. A target sequence in exon 3 (GCTCGACACAATGAGTCCAT) was selected for cloning. The target sequence was tested to be unique in the *C. elegans* genome (blast search on wormbase.org) to reduce the risk of off-target mutations. The target sequence was cloned into the pPD162 vector (*C. elegans* CRISPR/Cas9 vector from ref. 22) using the Q5 site-directed mutagenesis kit (New England Biolabs, E0554S). We used a forward primer including the target sequence (5′-ctcgacacaatgagtccatGTTTTAGAGCTAGAAATAGCAAGT-3′) and a general reverse primer (5′-CAAGACATCTCGCAATAGG-3′). Successful cloning was confirmed by sequencing at the site of integration as well as sequencing different sites of the vector.

Young adult wild type animals were injected with the CRISPR/Cas9 injection mix (50 ng/μl of pPD162 including the target sequence and 10 ng/μl of the CFP injection marker pPD136.61 (Fire Lab Vector Kit, 1999, linearised with ScaI) diluted in injection buffer^23^). The fluorescent injection marker was only used to control for successful injection. Single fluorescent progeny were isolated and checked for heterozygous mutations by sequencing and by the SURVEYOR^®^ mutation detection kit from Transgenomics. Single progeny of heterozygous mutants were further isolated on individual plates and analysed for homozygous mutations (SURVEYOR^®^ mutation detection and sequencing).

### Metabolite measurements

Age-synchronised animals were transferred to NGM medium containing 5-fluoro-2′ deoxy-uridine (FUDR) on day 1 of adulthood (day after larval stage 4) to prevent offspring from growing. On day 4 of adulthood, about 2000 animals/experimental group were washed off the plates with PBS and extensively washed to remove as much bacteria from cuticle and gut as possible. cOmplete^TM^ protease inhibitor (Roche) was then added and the samples were snap frozen. Only samples from the same experiment round were compared. The samples were lysed by sonication, excessive worm debris was removed and the protein concentration was determined. The protein concentration of the different samples was equalised and tryptophan and kynurenine levels were measured by LC-MS/MS as described before^8^.

### Immunodetection of TDO-2 in *C. elegans*

Lysates were generated as described under metabolite measurements. Proteins were denatured for 10 minutes at 70 °C. Proteins were separated by SDS-PAGE (25 μg total lysate per lane) and blotted to a PVDF membrane. The blot was blocked with 5% milk powder in PBS-Tween (0.1%). For immunodetection of TDO-2, we generated a *C. elegans*-specific polyclonal antibody in rabbits (Eurogentec, NL13061) against the peptide SEHSNLSHSQSSESD. The antibody was diluted 1:10,000 in 5% milk/PBST and binding took place at room temperature for 1 hour. To visualise the binding of the anti-TDO-2 antibody, a HRP-coupled anti-rabbit antibody (mouse) was used.

### Reproductive lifespan assay

Ten animals per condition were tested for their reproduction behaviour by transferring them every day to a fresh NGM plate. Eggs that were left behind could hatch and the number of hatched progeny was counted for the specific time period. Experiments were repeated and one representative graph is shown. Statistical analysis was by one-way ANOVA with post-hoc Bonferroni algorithm.

### Motility

Age-synchronised animals were grown on FUDR-containing NGM medium from day 1 of adulthood onwards to prevent the growth of the offspring. Swimming assays were performed on 15 animals per experimental group on different days of adulthood as indicated. For the assay, a single worm was placed in a drop of M9 buffer and was allowed to acclimate for 30 seconds before its swimming movements were counted for another 30 seconds. Experiments were repeated 3 times and one representative graph is shown. Statistical analysis was by two-way ANOVA with post-hoc Bonferroni algorithm.

### Lifespan experiments

100 animals per experimental group were scored for their lifespan behaviour. Worms were age-synchronised and transferred to FUDR-containing NGM medium on day 1 of adulthood to prevent offspring from growing. The different mutations of the experimental groups were blinded before starting the experiment. At each time point, living animals were counted and dead animals (no longer showing nose touch response) removed. Animals that disappeared during the assay were excluded from the analysis. Experiments were performed at 20 °C and were repeated five times. One representative curve is shown. Curves were generated using Graphpad Prism software. Statistical analysis was by Log-rank (Mantel-Cox) algorithm.

### Sequence alignment

The protein sequences for TDOs were retrieved from www.uniprot.org (H. *sapiens*: P48775, C. *elegans*: Q09474D. *rerio*: Q7SY53, D. *melanogaster*: P20351, X. *leavis*: Q5U4U6, M. *musculus*: P48776, R. *norvegicus*: P21643, accessed 18 Oct. 2016). Protein sequences were aligned using ClustalW2 or Clustal Omega. The default settings were applied (ClustalW accessed 18 July 2014, Clustal Omega 18 Oct. 2016)^24^. Haem and tryptophan binding sites were adopted from ref. 19.

### Homology models for wild type and mutant *C. elegans* TDO-2

BLAST was used to search for the TDO molecule that in terms of X-ray structure had the most homology to *C. elegans* TDO-2^25^. This turned out to be human TDO (ref. 20; PDB identifier 4PW8) with a sequence identity of 48.4%. On basis of this structure, models of the wild type and mutant proteins were built automatically using the SwissModel server^26^ and checked manually. In addition, we used the same crystal structure (4PW8) to model the structure of a human TDO where amino acids ALD were deleted at the equivalent position of PLD in the *C. elegans* enzyme. However, the human TDO X-ray structure (PDB identifier 4PW8) does not contain a haem moiety, which is critical for the catalytic activity of the enzyme. We therefore used the Coot program to superimpose the models of all TDO structures on the basis of secondary structures^27^. This demonstrated that the haem moiety of *X. campestris* TDO^19^ is perfectly positioned over the catalytic site of *C. elegans* TDO-2. This haem moiety was therefore added to our 3D models of wild type and mutant *C. elegans* TDO-2. All pictures were generated using Pymol software^28^.

## Additional Information

**How to cite this article**: Michels, H. *et al*. Identification of an evolutionary conserved structural loop that is required for the enzymatic and biological function of tryptophan 2,3-dioxygenase. *Sci. Rep.* **6**, 39199; doi: 10.1038/srep39199 (2016).

**Publisher's note:** Springer Nature remains neutral with regard to jurisdictional claims in published maps and institutional affiliations.

## Supplementary Material

Supplementary Information
