# Supplementary material for: Identification of an evolutionary conserved structural loop that is required for the enzymatic and biological function of tryptophan 2,3-dioxygenase
Source: Sci Rep. 2016 Dec 20;6:39199. doi: 10.1038/srep39199 (PMC5171515; doi:10.1038/srep39199)
Supplement: Supplementary Information [file srep39199-s1.pdf]

Supplementary Information

Identification of an evolutionary conserved structural loop that is required for the enzymatic and biological function of tryptophan 2,3-dioxygenase

Authors

Helen Michels[1], Renée I. Seinstra[1], Joost Uitdehaag[2], Mandy Koopman[1], Martijn van Faassen[3], Céline N. Martineau[1], Ido P. Kema[3], Rogier Buijsman[2], Ellen A.A. Nollen[1*]

Institutions

[1]European Research Institute for the Biology of Aging, University of Groningen, University Medical Center Groningen, Laboratory of Molecular Neurobiology of Aging, The Netherlands

[2]Netherlands Translational Research Center B.V., Oss, The Netherlands

[3]University of Groningen, University Medical Center Groningen, Department of Laboratory Medicine, The Netherlands

*Correspondence: e.a.a.nollen@umcg.nl

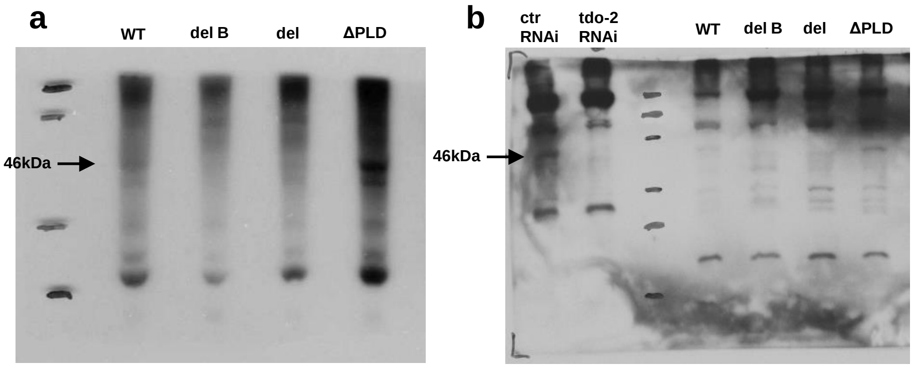

Supplementary Figure S1

(a) Full blot of figure 1b, 3 min exposure **(b)** Replicate blot**,** including tdo-2 RNAi validation of the antibody, 1min exposure

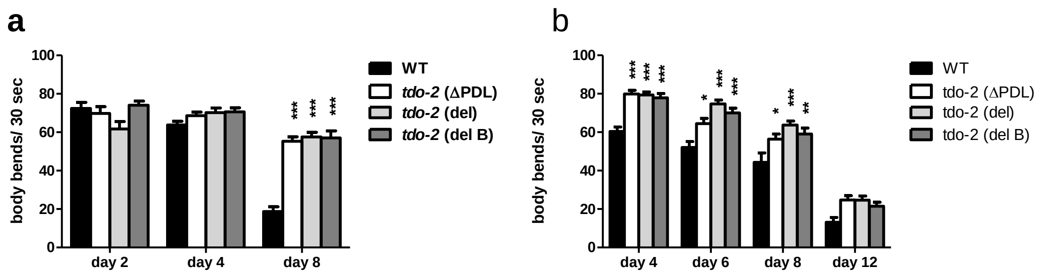

Supplementary Figure S2

(a) Motility replicate 1; Statistics, two-way ANOVA (Genotype, Time, Interaction: p <0.001) with post-hoc Bonferroni (compared to WT) **(b)** Motility replicate 3; Statistics, two-way ANOVA (Genotype: p <0.001, Time: p <0.001, Interaction: p =0.2572) with post-hoc Bonferroni (compared to WT) , Error bars in panels= SEM. Statistics for all panels (comparison with WT): * <0.05; ** <0.01; *** <0.001

Experiment number	Strain	Median	Mean ± SEM	% scored worms	versus	Median	Mean ± SEM	*P* value	% scored worms
1*	WT	22.0	20.16 +/- 0.38	91	*tdo-2* (ΔPLD)	19.0	20.26 +/- 0.52	0.4479	74
					tdo-2 (del)	22.0	21.08 +/- 0.44	0.0650	78
					tdo-2 (del B)	22.0	21.24 +/- 0.53	0.0024	71
2	WT	20.0	18.89 +/- 0.30	91	*tdo-2* (ΔPLD)	20.0	20.72 +/- 0.32	0.0002	92
					tdo-2 (del)	22.0	21.75 +/- 0.44	<0.0001	85
					tdo-2 (del B)	22.0	21.97 +/- 0.43	<0.0001	86
3*	WT	20.0	21.32 +/- 0.33	91	*tdo-2* (ΔPLD)	20.0	19.97 +/- 0.28	0.0022	72
					tdo-2 (del)	20.0	19.87 +/- 0.25	0.0006	67
					tdo-2 (del B)	20.0	19.84 +/- 0.21	0.0004	67
4	WT	20.0	19.66 +/- 0.35	95	*tdo-2* (ΔPLD)	20.0	20.82 +/- 0.34	0.0592	97
					tdo-2 (del)	18.0	18.60 +/- 0.31	0.0080	96
					tdo-2 (del B)	18.0	18.51 +/- 0.29	0.0021	94
5‡	WT	18.0	18.59 +/- 0.31	93	*tdo-2* (ΔPLD)	21.0	20.59 +/- 0.41	0.0001	89
					tdo-2 (del)	21.0	20.82 +/- 0.47	<0.0001	91
					tdo-2 (del B)	21.0	20.30 +/- 0.43	<0.0001	93

Supplementary Table S3

Summary of lifespan assays comparing wild type with tdo-2 mutated animals. * Experiments with more than 25% of the animals excluded because of rupture of the animals.

```
H. sapiens        MSGCPFLGNNFGYTFKKLPVEGSEEDKSQTGVNRA-SKGGLIYGNYLHLEKVLNAQELQS
R. norvegicus     MSGCPFSGNSVGYTLKNLSMEDNEEDGAQTGVNRA-SKGGLIYGDYLQLEKILNAQELQS
M. musculus       MSGCPFAGNSVGYTLKNVSMEDNEEDRAQTGVNRA-SKGGLIYGNYLQLEKILNAQELQS
C. elegans        -MACPYLGSGELTHRVT-FMEG--GEECQQGVNKVEMGFGQTYSEYLQLDKILTAQRLKS
D. melanogaster   -MSCPYAGNG--------------NDHDDSAVPLT-TEVGKIYGEYLMLDKLLDAQCMLS
D. rerio          MSGCPFLGGTLQLL-SSNPRQAEEEDGSQGGVNKA-AKGGIIYGDYLQLDKVLNAQVLQS
X. laevis         MSGCPFMAKKHHFTFSELSLEDKHEDNSQEGLNKA-SKGGLIYGDYLQLDKVLNAQELQS
                    .**:  .                          :  :  .:   .    *   *.:** *:*:* **  : *

H. sapiens        ETKGNKIHDEHLFIITHQAYELWFKQILWELDSVREIFQNGHVRDERNMLKVVSRMHRVS
R. norvegicus     EIKGNKIHDEHLFIITHQAYELWFKQILWELDSVREIFQNGHVRDERNMLKVMTRMHRVV
M. musculus       EVKGNKIHDEHLFIITHQAYELWFKQILWELDSVREIFQNGHVRDERNMLKVIARMHRVV
C. elegans        EADGQRVDDEHLFIVIHQAHELWFKQIIFDLDNVRKLLNN-TIVDETKTLKIVSGLDRMT
D. melanogaster   EEDKRPVHDEHLFIITHQAYELWFKQIIFEFDSIRDMLDA-EVIDETKTLEIVKRLNRVV
D. rerio          EQKGNKIHDEHLFIVTHQAYELWFKQILWELDSVRDLFIKKHVRDERNMLKVVSRIHRIT
X. laevis         EKKGNKIHDEHLFIVTHQAYELWFKQILWELDSVREIFQNGHVRDERNMLKVVARIHRIS
                  *  .  . :.******: ***:*******::::*.:*.::     : ** : *:::  :.*:

H. sapiens        VILKLLVQQFSILETMTALDFNDFREYLSPASGFQSLQFRLLENKIGVLQNMRVPYNRRH
R. norvegicus     VIFKLLVQQFSVLETMTALDFNDFREYLSPASGFQSLQFRLLENKIGVLQSLRVPYNRKH
M. musculus       VIFKLLVQQFSVLETMTALDFNDFREYLSPASGFQSLQFRLLENKIGVLQSLRVPYNRKH
C. elegans        KILSLLTEQITLLDTMSPLDFVDFRKYLTPASGFQSLQFRVLENKLGVRQERRIKYNAQH
D. melanogaster   LILKLLVDQVPILETMTPLDFMDFRKYLAPASGFQSLQFRLIENKLGVLTEQRVRYNQK-
D. rerio          MIFKLLVDQFAVLETMTALDFFDFREYLSPASGFQSLQFRLLEQKIGVADHLRVPYNRRH
X. laevis         MILKLLVEQFSVLETMTALDFFDFRDYLSPASGFQSLQFRLLENKIGVPEILRVPYNRRH
                   *:.**.:*. :*:**: :** ***.**:***********::*:*:**     *: ** :

H. sapiens        YRDNFKG-EENELLLKSEQEKTLLELVEAWLERTPGLEPH--GFNFWGKLEKNITRGLEE
R. norvegicus     YRDNFEG-DYNELLLKSEQEQTLLQLVEAWLERTPGLEPH--GFNFWGKFEKNILKGLEE
M. musculus       YRDNFGG-DYNELLLKSEQEQTLLQLVEAWLERTPGLEPN--GFNFWGKFEKNILKGLEE
C. elegans        YKNVFNDTD-LKTLNVTEEEKSLLTLIESWLERTPGLKSTSEDEGFWIKYEKSVNKYLAD
D. melanogaster   YSDVFSDEEARNSIRNSEKDPSLLELVQRWLERTPGLEES--GFNFWAKFQESVDRFLEA
D. rerio          YRDNFHG-EESETLLSSEQEPTLLQLVEQWLERTPGLEKD--GFNFWGKLQANIEEGLKR
X. laevis         YRDNFKG-ETNELLLRSEQEPTLLGLVEAWLERTPGLEEE--GFHFWGKLEVNIFRALEE
                  *  : *  .  :   : :  :*:: :** *:: ********:    .  ** * : .: . *

H. sapiens        EFIRIQAKE-ESEEKEEQVAEFQKQKEVLLSLFDEKRHEHLLSKGERRLSYRALQGALMI
R. norvegicus     EFLKIQAKK-DSEEKEEQMAEFRKQKEVLLCLFDEKRHDYLLSKGERRLSYRALQGALMI
M. musculus       EFLRIQAKT-DSEEKEEQMAEFRKQKEVLLCLFDEKRHDYLLSKGERRLSYRALQGALMI
C. elegans        LAKQAADPSNTEEIAKQLTAEYHKTADAFQSILDPRQHEQHIRNGNRLLSHDATKGAMMI
D. melanogaster   QVQSAMEEP-VEKAKNYRLMDIEKRREVYRSIFDPAVHDALVRRGDRRFSHRALQGAIMI
D. rerio          EKHQVEKME-DTEVKQELLEDLNKQMETFTALFDSKRHEHLLSKGERRLSYKALQGALMI
X. laevis         ELQAAKTKP-DSEDKEEHLAELQKQKELFGALFDERRHEHLLSKGERRLSYKALKGALMI
                              :  :    :  .*  :   .::*   *:  :  .*:*  :*: * :**:**

H. sapiens        YFYREEPRFQVPFQLLTSLMDIDSLMTKWRYNHVCMVHRMLGSK-AGTGGSSGYHYLRST
R. norvegicus     YFYREEPRFQVPFQLLTSLMDIDTLMTKWRYNHVCMVHRMLGSK-AGTGGSSGYYYLRST
M. musculus       YFYREEPRFQVPFQLLTSLMDIDTLMTKWRYNHVCMVHRMLGTK-AGTGGSSGYHYLRST
C. elegans        YFYRDMPRFSQPYQILTFLMDIDSLFTKWRYNHVLLVQRMLGAK-QGTGGSSGYMYLRST
D. melanogaster   TFYRDEPRFSQPHQLLTLLMDIDSLITKWRYNHVIMVQRMIGSQQLGTGGSSGYQYLRST
D. rerio          NFYREEPRFQVPFQLLTALMEIDTLMTKWRYNHVCMVHRMIGSK-AGTGGSSGYHYLRST
X. laevis         NFYREEPRFQVPFQLLTSLMEIDTLMTKWRYNHVCMVHRMIGSK-AGTGGSSGYQYLRST
                   ***: ***. *.*:** **:**:*:********  :*:**:*::   ******** *****

H. sapiens        VSDRYKVFVDLFNLSTYLIPRHWIPKMNPTIHKFLYTAEYCDSSYFSSDESD--
R. norvegicus     VSDRYKVFVDLFNLSSYLVPRHWIPKMNPIIHKFLYTAEYSDSSYFSSDESD--
M. musculus       VSDRYKVFVDLFNLSTYLVPRHWVPKMNPIIHKFLYTAEYSDSSYFSSDESD--
C. elegans        VSDRYKVFLDLFNLSTWLIPREYIPMLSPRMVKTLSEHSNLSHSQSS--ESD--
D. melanogaster   LSDRYKVFLDLFNLSTFLIPREAIPPLDETIRKKLINKSV--------------
D. rerio          VSDRYKVFVDLFNLATFLVPRSWVPKLNPNIHKFPYTAECYDSSYNSCSSEDSD
X. laevis         VSDRYKVFVDLFNLSTYLVPRHWVPRLNPSIHKFLYTAECCDSSYFSSDDSD--
                  :*******:*****:::*:**  :* :.  : *     .
```

Supplementary Figure S4

Multiple sequence alignment of TDO; yellow = tryptophan binding site, red = haem binding site, orange = binding site for haem and tryptophan, blue = residues missing in mutant *tdo-2 (ΔPLD)*.
